# Abrasive Sensitivity of Martensitic and a Multi-Phase Steels under Different Abrasive Conditions

**DOI:** 10.3390/ma14061343

**Published:** 2021-03-10

**Authors:** Ádám Kalácska, László Székely, Róbert Zsolt Keresztes, András Gábora, Tamás Mankovits, Patrick De Baets

**Affiliations:** 1 Soete Laboratory, Department of Electromechanical, Systems and Metal Engineering, Ghent University, Technologiepark 46, B-9052 Zwijnaarde, 9000 Ghent, Belgium; Patrick.DeBaets@UGent.be; 2 Flanders Make—The Strategic Research Centre for the Manufacturing Industry, 3001 Leuven, Belgium; 3 Institute of Mathematics and Basic Science, Szent István Campus, MATE, Páter Károly u. 1, 2100 Gödöllő, Hungary; Szekely.Laszlo@uni-mate.hu; 4 Institute of Technology, Szent István Campus, MATE, Páter Károly u. 1, 2100 Gödöllő, Hungary; Keresztes.Robert.Zsolt@uni-mate.hu; 5 Department of Mechanical Engineering, Faculty of Engineering, University of Debrecen, Ótemető u. 2-4, 4028 Debrecen, Hungary; andrasgabora@eng.unideb.hu (A.G.); tamas.mankovits@eng.unideb.hu (T.M.)

**Keywords:** steel, abrasive wear, surface analysis, mechanical properties, regression model

## Abstract

The wear behaviour of two martensitic and one multiphase steel targeted for abrasion and erosion applications in agriculture and mining industry were investigated in three abrasive test systems with different complexity. Scratch tests were performed with different indenter radii, shapes, and loads. The material behaviour was also investigated in multi-asperity contact systems. Pin-on-disc tests were performed with various loads and abrasive particles, as well as abrasive slurry-pot tests with different sliding velocities, distances, and impact angles of the abrasive media were performed. Comparing the test systems, the tested materials ranked similarly based on their wear performance, however, in each configuration, the dominant variable of the wear mechanism differed. The significance and contributions of test paramecenterters, the material’s mechanical properties (*H*, $\sigma_{M}$, $\sigma_{Y}$, *E*, $\varepsilon_{M}$, $\varepsilon_{B}$, *W*, $\sigma_{c}$, $E_{c}$) and the dimensionless numbers formed from them were investigated on the wear behaviour and the surface deformation. Correlation between parameters was established by multiple linear regression models. The sensitivity of the tested materials to abrasion was evaluated taking into account the wide range of influencing parameters.

## 1. Introduction

In engineering applications, where harsh operating conditions prevail such as in mineral industry, soil processing, and agriculture, tribological investigation of the operational variable dependent, location-specific wear and damage mechanisms are particularly important [1,2]. The demand of extended lifetime for machine elements and an increase in cost efficiency urges developing new materials, and the most cost-effective wear-resistant materials should be identified and applied [3]. In 1986, the total annual cost due to wear in the agricultural sector of Canada reached around $940 million [4]. In agricultural machinery wear parts are frequently produced from specific steels, e.g., tillage tools, which are exposed to abrasive wear are made of 27MnB5 [1]. Other components such as slurry pumps, extruders, pipes carrying ores, and coal slurry nozzles are exposed to slurry abrasive wear in power plants and mineral processing industries [5,6]. The equipment and components used in slurry transport experience multiple wear modes and damage mechanisms in the form of abrasion, slurry erosion, and corrosion. [7,8]. Straight pipes are good examples of abrasion caused by sliding and rolling particles [3]. These conditions can also be encountered in agricultural applications where crop transport (e.g., wet paddy rice) and cultivation can cause similar problems [9]. The agricultural and mining machine components operate in a wide range of conditions that shape the abrasive wear. The harmonization of environmental and technical requirements is a complex aspect in the selection and development of the applied structural materials. Therefore, in abrasion areas sometimes light and corrosion-resistant polymers [10] or aluminum alloys are used, even when foamed [11], but where the presence of mass and strength is essential (e.g., tillage and cultivators), the use of different steels is preferred. Generally, alloyed martensitic steels are utilized in these applications, often with hard alloy coatings to aid the wear resistance [12].

Surface damage to a tribological pair is usually caused by consecutive small steps induced by wear micro-mechanisms. At the micro-level, the four different modes of abrasion are micro-ploughing, where the material is plastically deformed and displaced, micro-cutting, defined as micro-machining with chip removal, micro-fatigue, and micro-cracking [13]. The micro-mechanisms generate physical modification at the surface whether or not material removal happens. In this way, the resulted surface damage on the material is the summarized outcome of one or more micro-mechanisms leading to macroscale damage in the form of grooves caused by abrasive particles [14]. In accordance with the tribo-system characterization, the experienced macro-wear is the result of a complex tribo-system response influenced by the testing conditions besides the materials in contact [15]. To select the best candidate material to optimize a tribological system, the tribosystem elements (contacting materials, their geometry and surface topography, the configuration of contact, the relative motion, the loading condition, and the environment with possible intermediates) need to be investigated [13].

The accurate prediction of abrasion behaviour of the materials is difficult as numerous parameters govern the abrasion phenomenon. Researchers concluded that the abrasive wear rate within a wear regime is usually inversely proportional to the hardness of the abraded body [14,16,17]. Proportional relation was found to the normal load [18,19,20,21], and the sliding velocity [18,22]. Effect of particle size [18,23,24,25], shape [16,17], and the type [18] of abrasive media on the wear have been studied. These findings were also confirmed by pin-on-disc testing of different abrasion-targeted steels with different microstructures including ferritic stainless steel, medium alloyed ferritic carbon steel, and medium alloyed martensitic carbon steel [2]. The basic relationships between material properties and specific wear have been investigated in numerous works of research, however the involved material properties are often limited to tensile properties besides the hardness. Quite a few materials have been considered to be used for abrasive slurry transport [3], but their wear resistance may vary according to the system properties and possible newly developed materials have not been investigated. Furthermore, testing is often done in standardized test set-ups, where connection to a specific application is limited. Due to the different abrasion forms that can be present in a wide range, the present investigation is expanded to multiple test systems, which was not connected in the literature previously.

Multiple research was performed to estimate the abrasion resistance based on well-defined mechanical properties, e.g., Vickers hardness [14,26,27], ultimate tensile strength and yield strength [17,28,29,30], and uniform elongation [31]. Non-linear connections have been found between the abrasive wear resistance and the hardness along with the tensile properties for multi-phase low alloyed steels [17,32,33]. The reported contradictory dependencies against basic mechanical properties were explained with limited data sets, imperfect separation of microstructural and compositional effects, an overly simplified statistical analysis, the use of different testing methods, or the absence of a unique correlation [15]. The abrasive wear resistance of wall lining materials in iron ore mining was investigated [34]. The wear rate was found to have a proportional relationship to the Knoop hardness values, therefore the hardness tests could be used to represent the abrasive wear ranking of the studied lining materials. The wear behavior of 7075 and 7075/5 wt% Al_2_O_3_ alloyed composites was investigated with a focus on microstructure, density, and hardness, produced by powder metallurgy [35]. Analysis of variance (ANOVA) highlighted the percentage contribution of particle size (11.48%), sliding velocity (0.6%), and applied load (86.9%) on the wear. The experimental data of this work were correlated by reference to dimensional analysis considerations followed by least-squares polynomial regression [36]. The influence of particle impact velocity, density, concentration, and hardness on the erosion wear was identified, however the effect of the impact angle was neglected. Dry sliding wear behavior of composites was investigated using a pin-on-disc set-up with different loads (10 N, 20 N, 30 N, 40 N), sliding velocity and wt% of MoB (1–4) as variables [37]. ANOVA was used to study the significance and influence of parameters on the material loss, and the correlation between parameters was obtained by a regression equation. MoB content of the composites was observed to be the most dominant factor (57.09%), followed by sliding velocity and load. A broad investigation was performed on low alloyed steels to analyze the connection between abrasion resistance and standard mechanical properties of the materials. [15]. ASTM G65 standard abrasion test was used for 20 chemically identical steel samples and 20 with different chemical compositions and microstructure but with similar mechanical properties. A strong linear connection between the wear performance and some mechanical properties was found for the chemically identical samples, however the samples with similar mechanical properties showed weaker correlations. In the recent research work by Bustillo et al. [38], artificial intelligence models were used to forecast the surface wear based on the surface isotropy levels. For mass loss, the radial basis networks (RBFs) method resulted in the most precise prediction, whereas for surface deformation, the multilayer perceptrons (MLPs) technique brought the best results in terms of indicator Ra. Matuszewski et al. [39] developed mathematical models based on experimental results to describe the connection of the surface wear and the studied influencing factors (e.g., load, velocity, surface parameters). The output parameters included mass loss, change in geometry, as well as surface roughness parameters. Their study enabled to predict the wear process of kinematic pairs with conformal contact.

Numerous investigations can be found in the literature, where the researchers connect the abrasion resistance of steels to their standard mechanical properties as determined in simple tensile and hardness testing [15]. However, many unclear or even contradictory correlations have been found, partly due to small data sets, distinct testing methods, and relatively straightforward data processing techniques. A comprehensive evaluation of the abrasion sensitivity in martensitic steels for different test systems—taking into account tensile, hardness, impact and compressive mechanical properties and the dimensionless numbers that can be formed from them—is not in the literature yet, but that could support the proper steel material selection and developments for a given application.

Understanding the material behavior under different abrasive wear conditions is an important aspect from a design point of view to propose better-performing wear-resistant machine parts. To determine the wear performance, further laboratory tests are required to cover a wide range of abrasion processes in different tribo-systems. Taking the different abrasion forms that can be present in a wide range into consideration, our investigation is expanded to multiple test systems. Correlation analyses were performed to investigate the connection of abrasive wear features with extended mechanical property combinations (hardness *H*, ultimate tensile strength *σ_M_*, yield strength *σ_Y_*, Young modulus *E,* uniform elongation *ε_M_*, fracture elongation *ε_B_*, Charpy ISO 148-1 *W*, compressive strength *σ_c_*, compressive modulus *E_c_*) in single- and multi-asperity tests systems and also in a slurry containing system, which can model different abrasive modes on the surfaces. Single-asperity scratch tests were performed with various loads, indenter tip radius, and attack angle in order to study the abrasive scratch-resistance of the materials. Pin-on-disc tests were used with various loads, sliding distance, and abrasive particles to investigate the material response in a multi-asperity contact abrasion process. The material behavior was further studied in a slurry-abrasion test system with different sliding velocity, distance, and impact angle of the abrasives. Large amounts of measured tribo data were processed by multiple linear regression models using IBM SPSS 25 software to determine and evaluate the abrasive sensitivity of wear performance and the change of 3D surface topography to material properties and to test system characteristics. In this way, not only was the wear performance of the materials compared in the different test systems, but the significance and contributions of parameters on wear behaviour were studied. A correlation analysis was performed on the processed data between abrasion resistance and extended mechanical properties by regression models. The abrasion sensitivity of the tested materials was evaluated considering a broad range of influencing parameters.

## 2. Materials and Methods

As mentioned in the introduction, three test methods have been used for a broad study about abrasion resistance of the selected abrasion targeted steels: A scratch test, an abrasive pin-on-disc test, and a slurry pot system. The resistance of a material to abrasion by one single indenting point is measured using single-asperity contact scratch testing [40]. It simulates an ideal scenario in controlled conditions, where a single pass indenter abrades on a clean surface. However, this scenario is rarely encountered in real applications. As previously reported [41], the wear rates determined only from single scratch tests are not a proper representation of the true behavior of the steel in high-stress abrasion conditions. In the multi-asperity contact abrasive pin-on-disc, the contact surface of the steel samples interacts with new SiC particles during the sliding resulting in surface deformation and wear of the specimen. The mentioned wear process was found to be dominant in case of agricultural tines and cultivator elements operating below 5 m/s [1]. Abrasive slurry erosion was accomplished in the slurry pot system, which is often encountered in agriculture (e.g., when wet, soil contaminated crops are harvested, and in mining in the environment of abrasive slurry (e.g., slurry pipes).

### 2.1. Materials

In this investigation, the wear performance of 3 materials was studied. Two different abrasion-resistant martensitic steels with the same chemical composition but different post-processing. After hot rolling, material “FM” is water quenched and coiled at room temperature, resulting in a fresh martensitic structure. Reheating followed by air cooling results in a low tempered martensitic structure of material “TM”. The third tested material was a more ductile multiphase steel (material “MP”), characterized by martensitic structure along with small fractions of retained austenite. The chemical composition of all tested materials is shown in Table 1.

The characteristic features and properties of the three materials are summarized in Table 2.

### 2.2. Abrasive Scratch Test

**Figure 1:**
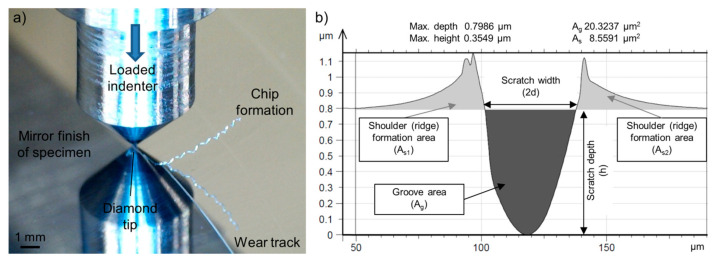
(**a**) Scratch testing (MP material, F_N_ 10.8 N, r 25 µm, γ 90°); (**b**) Extracted groove data.

The steel specimens for the single-asperity scratch test were processed through grinding and polishing following ASTM G171—03(2017). The surface topography of the prepared steel samples was investigated in unworn and post-mortem conditions using a non-contact optical profilometer (Taylor Hobson CCI HD, Leicester, UK). Rockwell-type diamond indenters were used to create the scratches with different indenter geometry and load conditions on a modified pin-on-disc test-rig. Figure 1a shows a scratch process of the loaded indenter engaging the polished specimen surface and removing material through micro-cutting mechanism.

TalyMap software (version 6.2, Digital Surf, Besançon, France) was used to extract the 3D surface data of the wear track. Multiple cross-section profiles were processed along the wear track from defined locations (2 profile/µm). The derived data were then merged into one cross-section profile to study, as seen in Figure 1b.

The wear micro-mechanisms were identified with the degree of penetration (Dp) of the wear groove, which was calculated from the averaged cross-section profile after the scratch tests. Dp not only serves as a tool to identify the micro-abrasion processes from the wear track geometry but also gives an indication about the wear severity [42]. From the literature [43], the Dp is calculated by dividing the groove depth (*h*) (µm) with the half-width of the profile at the surface level (d) (µm).

As it can be seen in Figure 1b, the value of the groove area (*A_g_*), as well as the shoulder/ridge areas (A_s1_, A_s2_) are derived from the software. In order to get the volume of the groove (*V_g_* (mm^3^)) and of the ridges/shoulders (*V_s_* [mm^3^]) the values are multiplied with the scratch length. The wear volume is the difference between the shoulder volume to the groove volume. Different Dp value ranges are associated with each wear micro-mechanism. The material hardness influences these ranges. The transition in micro-mechanism from micro-ploughing to micro-cutting is induced by increasing the attack angle of the abrading particle, hence increase of Dp [17,44].

To compare the wear performance of the tested materials, the specific wear rate *k* (mm^3^/Nm) was calculated according to the literature [45]:

(1) $$k=\frac{V_{g}-V_{s}}{V_{g}}\cdot \frac{V_{g}}{N\cdot s},$$

where *N* is the normal load (N) and s is the sliding distance (m).

In the abrasive scratch tests, where single-asperity contact between the loaded indenter and the material surface induces the deformation, test variables of normal load (0.9 N, 1.3 N, 5.8 N, 10.8 N), attack angle (30°/45° corresponding to cone angle 120°/90°), and indenter tip radius (25 µm, 50 µm, 100 µm, 200 µm) for all the three different steel grades were used. A constant sliding speed of 0.45 mm/s was applied throughout the 30 mm long scratch. Tests were repeated three times for extreme and intermediate test conditions.

### 2.3. Pin-on-Disc Test

**Figure 2:**
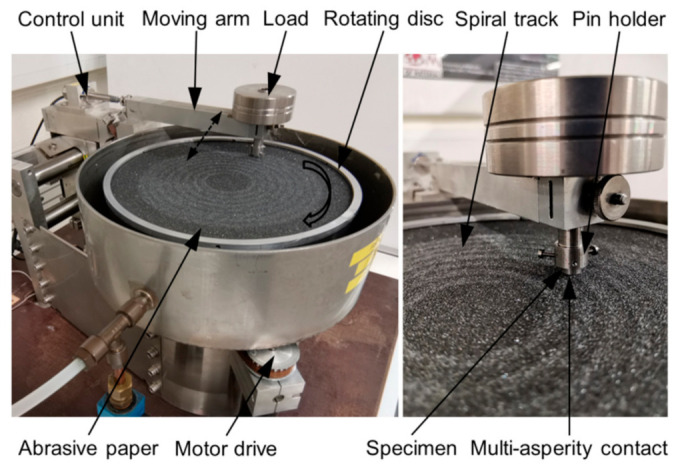
Abrasive pin-on-disc testing set-up.

Pin-on-disc tests were performed according to ASTM G132 [46] multi-asperity testing. The test set-up (developed at Labo Soete, Gent, Belgium) is shown in Figure 2.

As confirmed from the literature [47], the machined surface structure isotropy affects the progress of material removal, however the same material processing and specimen preparation was used for this investigation, which enables the comparison of the materials. Cylindrical pin samples (diameter 8mm and height 5 mm) were produced from steel sheets using laser cutting technology. The uniform specimen preparation method included the processing of the contact surface with P80 sandpaper to a sliding distance of 3 m under 12 N load, followed by polishing with P800 with the same settings. After the mechanical finishing, an ultrasonic cleaning followed, before checking the pre-test 2D surface topography. The final surface state was characterized perpendicular to the one-way oriented surface grooves, with Ra ~ 1 µm average Rz of 8.3 µm, Rq of 1.6 µm. In the test system, four types of SiC abrasive papers were applied (P80—200 µm, P120—100 µm, P180—50 µm, P800—25 µm) introducing different average attack angles (P80—28°, P120—32°, P180—35°, P800—45°) [48]. In the pin-on-disc tests, the multi-asperity contact between the loaded specimen and the used different abrasive papers under relative motion at 100 mm/s invoked the material removal. The material loss was tracked by an electronic balance with an accuracy of 0.1 mg. Samples were measured in unworn condition and after every 3 m sliding. Keyence VR 5200 microscope (Keyence, Osaka, Japan) was used to examine the 3D worn surface topography. Furthermore, the post-mortem steel samples were checked for their Vickers hardness on the contact surface at 10 distinct locations. The sliding distance up to 9 m, the applied normal loads (4.5 N, 11.4 N, 17.3 N, 24.2 N), and the abrasive particle size and attack angle were considered as test variables. For the extreme and intermediate cases, three replicates were performed. The ambient conditions were registered as the following: 21–23 °C, 45–55% RH.

### 2.4. Slurry-Pot Test System

In a more complex multi-asperity contact test system, the specimens were tested in abrasive slurry. The test configuration was designed to experimentally simulate the wear of components that experiences abrasive erosion wear mechanisms. This test set-up enables to test in a more complex environment closer to the real application, where co-existing wear mechanism occurs e.g., on agricultural tines [1]. Although more uncertainties are involved in the slurry-contact mechanism, the effects of the controlled wear-influencing parameters are investigated. The method also provides an efficient ranking of materials in terms of wear rate.

**Figure 3:**
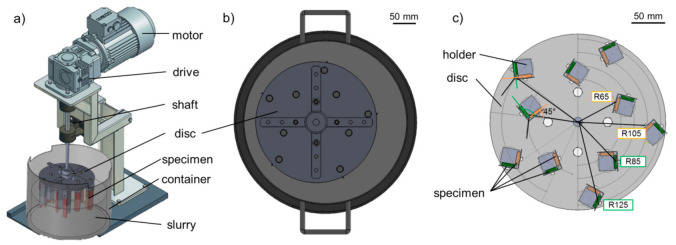
(**a**) Test rig concept [50]; (**b**) top view of the eccentric placed disc in pot; (**c**) bottom view of the disc with the mounted specimen on radii 65, 85, 105, 125 mm in different angle configuration.

The concept of the test rig is shown in Figure 3a. The test set-up was developed and manufactured in MATE, Gödöllő, Hungary. The rotating disc is driven by a three-phase induction motor on a vertical shaft through a 1:10 worm drive. The shaft is supported by ball bearings. The disc is eccentrically placed in a cylindrical container (Figure 3b) to provide better mixing of the media in the container, which is accommodated with slurry formed from abrasive media and water in a 4:1 ratio.

The used abrasive medium was corundum (Korund EKF-10, MOTIM, Mosonmagyaróvár, Hungary). This is a crystalline form of Al_2_O_3_, a rock-forming mineral. The average size of a new corundum particle is in the range of 2000–2360 µm, with 3.87 kg/dm^3^ density and 9.0 Mohs hardness/2050 Knopp kN/mm^2^. The effectiveness of an abrasive depends on its hardness, shape, grain density, and grain size. The smaller the grains, the slower their effect, but the angular shape results in increased abrasion [49].

On the disc, vertically mounted specimen holder columns are placed on two different radii (75, 115 mm), five pieces on each radius. Figure 3c shows the bottom view of the disc with the mounted holders and specimens in different configurations. On each specimen holder column, two specimens are mounted perpendicular to each other. These holders could be rotated around their axis to set the orientation angle of the specimen with respect to the slurry flow direction. Due to the geometry of the specimen holders, the center of the specimens was placed on four different radii (65, 85, 105, 125 mm). The samples on radius 65 mm and 105 mm were facing the center of rotation (center shaft), and the samples on radius 85 mm and 125 mm were facing the pot wall. The top 20 mm of the specimen—where they are mounted to the specimen holder—is protected with an extra plate to keep a reference, unworn zone on each sample. In static position, the specimens were covered in the slurry to a depth of 60 mm depth (half of the specimen height).

During operation, the rotation of the shaft agitates the slurry and causes the moving specimen to slide and impact against the abrasive in the slurry. Depending on the location of the samples on the holder, collisions occur with different impact mean velocity values and in different angles. Tests were performed at a rotational speed of 140 rounds/min and a room temperature of 21 ± 1 °C. The slurry pot was placed inside a container with a continuous flow of cooling water, providing the cooling through the pot walls.

In the developed abrasive slurry test configuration, the effect of sliding distance, sliding velocity, and collision impact angle on the material loss was investigated. The change in surface topography and hardness was also monitored. Slurry pot testing of 20 specimens was carried out for 9 × 20 h of operation. All three materials were tested with the same conditions, and the cycle was repeated to ensure three repetitions for each material. The testing parameters and conditions are shown in Table 3.

At every 20 h intermediate pause, the slurry was replaced with fresh abrasives, and the samples were investigated for their weight and hardness of the worn zone. After cleaning the specimens, the surface roughness was monitored with Mitutoyo Surftest SJ 211 (Kawasaki, Japan) stylus 2D profilometry. The hardness was measured with Zwick (Ulm, Germany) Roell Indentec 81,875 A/B tester using a diamond tip indenter and 30 kg (~300 N) indentation force. On all specimens, 10 indents were in the worn zone and on the unworn reference zone. After 180 h of testing the specimen were investigated with Keyence (Osaka, Japan) VR-5200 wide-area 3D microscopy to analyze the worn specimen surface topography change caused by wear.

### 2.5. Evaluation Methodology

In the single-asperity scratch test system, the wear groove characteristics (groove width *2d* (µm), groove depth *h* (µm), groove area *A_g_* (µm^2^), ridge/material shoulder area *A_s_* (µm)) were registered under 32 system conditions (different normal loads F_N_ (N), different indenter cone angle γ (°), different indenter tip radius *r* (µm)) in the function of sliding distance, *s* (m). A specific wear rate was calculated (wear volume normalized with load and sliding distance) (mm^3^/N·m) for the material ranking.

In the pin-on-disc test system: Wear as mass loss [g], worn surface hardness was registered under 16 system conditions (different normal loads F_N_ (N), different abrasive size *d* (µm) with different attack angle *α* (°)) as a function of sliding distance, *s* (m). Materials were ranked based on the wear (g/km).

In the slurry-pot system, to track the wear evolution, the mass (g) of the steel specimen was registered after 20 h of testing, which corresponds to one-ninth of the total sliding path. The relative wear (%) has been calculated and normalized to sliding distance (km). The results were compared in function of the defined impact angle *α* (°) and circumferential velocity *v* (m/s). The change of the worn surface hardness was also tracked in the function of the sliding distance *s* (m).

For both multi-asperity contact test systems, the surface topography was investigated before testing and in post-mortem condition. The following 2D surface parameters were extracted (ISO 4287): Ra (µm), Rz (µm), Rp (µm), Rv (µm), Rt (µm), Rc (µm), Rq (µm), Rsm (µm), Rsk [-], Rku [-].

The measured data were investigated as a function of the properties of the examined steels (Table 2) and the dimensionless numbers formed from these properties. The combined or derived dimensionless numbers are as follows:

1. $\frac{H}{E}$ the ratio between hardness and elasticity modulus,
2. $\frac{\sigma_{M}}{\sigma_{Y}}$ the ratio between ultimate tensile strength/yield strength,
3. $\frac{\varepsilon_{B}}{\varepsilon_{M}}$ the ratio between fracture elongation/elongation at maximum tensile load,
4. $\frac{W_{\left(20\right)}}{W_{\left(-40\right)}}$ (=*W*) the ratio between Charpy impact strength at 20 °C/Charpy impact strength at −40 °C,
5. $\frac{\sigma_{Y}E}{\sigma_{M}H}$ the ratio between combined tensile performance/combined bulk-surface stiffness,
6. $\frac{E_{c}}{\sigma_{c}}$ the ratio between compression modulus/compression strength,
7. $\frac{H\varepsilon_{B}}{\sigma_{Y}}$ the ratio between combined Hardness-strain capability/yield strength,
8. $\frac{\sigma_{Y}}{\sigma_{c}\varepsilon_{B}}$ the ratio between yield strength/combined compression—strain capability,
9. $\frac{WEH}{\sigma_{M}\sigma_{Y}}$ the ratio between combined Charpy ratio and strain capability/combined tensile strength,
10. $\frac{\varepsilon_{M}}{\sigma_{Y}}$ the ratio between elongation at maximum tensile load/yield strength,
11. $\frac{W}{\varepsilon_{B}}$ the ratio between Charpy impact strength ratio/fracture elongation,
12. $\frac{W\sigma_{Y}}{E}$ the multiplication of combined Charpy impact strength ratio and elastic tensile behaviour.

The statistical analysis relies on multiple linear regression models. Such models are useful if one examines how a dependent variable depends on several independent variables at one time, assuming that the dependence is (approximately) linear. Let n be the number of independent variables then the formulization of such model is

(2) $$Y=a_{0}+a_{1}X_{1}+a_{2}X_{2}+\ldots +a_{n}X_{n},$$

where Y is the dependent variable and $X_{1},X_{2},\ldots ,X_{n}$ are the independent variables. For a more detailed description of such models (in material science as well) see e.g., the authors’ previous paper [10]. The statistical evaluation of the models was carried out by using IBM SPSS 25. For our models, the stepwise method was used to enter a new variable into a specific linear regression model.

## 3. Results and Discussion

### 3.1. Scratch Test

**Figure 4:**
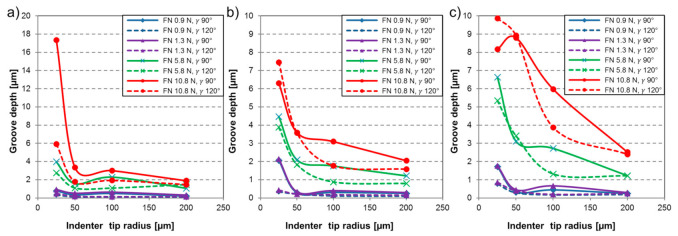
Depth of wear grooves [µm] in function of indenter tip radius [µm] with various applied load (F_N_) and indenter cone angle (γ) for material (**a**) FM, (**b**) TM, (**c**) MP.

The wear grooves were analyzed (groove depth and width as well as groove and ridge areas) to calculate the Dp and the specific wear rate of the materials. Figure 4 shows the depth of the wear grooves as a function of the indenter geometry and the applied load. Testing with higher loads evidenced in deeper grooves, however an increase in the tip radius resulted in the opposite effect. The cone angle was also found to have an inverse relation to the depth of the wear track. Hence, decrease in the attack angle generally created less-deep grooves, except for tests on the highest load with 25 µm tip radius in the case of TM and MP steel. The sharpest indenter (25 µm tip radius with 90° cone angle) with 10.8 N normal load provoked the highest stress concentration, leading to the deepest wear groove. Overall, the MP steel experienced the deepest scratches compared to the other investigated materials under the same testing conditions, which forecasts an inferior wear performance. It was reported in the literature [27], that lower material hardness promotes more severe material removal with deeper wear grooves with increased degree of penetration. To confirm this observation, the Dp values of the wear grooves were investigated.

**Figure 5:**
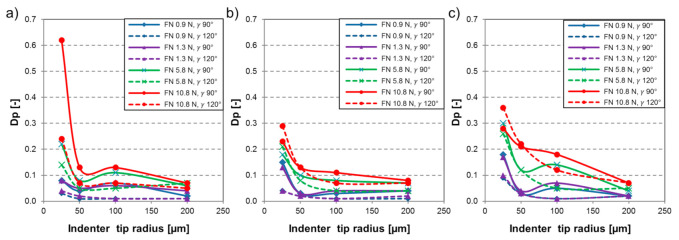
Degree of penetration [-] of wear grooves in function of indenter tip radius (µm) with various applied load (F_N_) and indenter cone angle (γ) for material (**a**) FM, (**b**) TM, (**c**) MP.

Figure 5 shows the Dp as a function of the indenter geometry and various applied normal loads. Dp range of 0–0.1 specifies that only plastic deformation of the material takes place through micro-ploughing. Above this zone, a transition happens with wedge formation to micro-cutting, where the material is removed, and chip is generated.

Overall, the MP material experienced wear grooves with elevated degree of penetration values compared to the martensitic steels. The sole exception is the test on FM material with the sharpest indenter (r 25 µm, γ 90°) and 10.8 N normal load (F_N_). The applied load was found to have a proportional relation with the Dp. Grooves characterized with low degree of penetration were reported [25] to represent plastic deformation with features of ridge formation along the wear track. In this way, test results on ~1 N load levels were characterized as micro-ploughing. A transition to micro-cutting was evident when testing with 5.8 N normal load. Further increase of the applied load resulted in more deep penetration of the indenter leading to elevated Dp and material removal through micro-cutting wear micro-mechanism. The influence of the indenter geometry was analyzed. The effect of the attack angle on the wear mechanism was previously investigated [51]. A proportional relation was confirmed between the attack angle, in general, the decrease in attack angle reduced the Dp.

**Figure 6:**
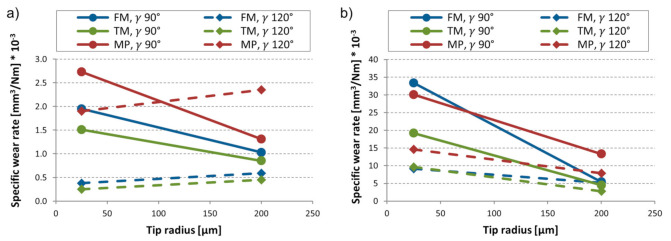
Specific wear rate of the tested materials (FM, TM, MP) in the function of tip radius (µm) and indenter cone angle (γ) for different loads (**a**) F_N_ 0.9 N, (**b**) F_N_ 10.8 N.

The material volume loss was normalized with the load and the sliding distance to get the specific wear rate, which is plotted against the tip radius in Figure 6. Concerning wear resistance, the best performing material according to the single-asperity tests was the TM—tempered martensitic material, followed by FM—fresh martensitic. The MP material was prone to micro-cutting and suffered more significant material loss. The distinct wear performance of the investigated martensitic and MP materials could be interpreted as a consequence of the different material hardness (Table 2). Comparing the martensitic materials, the initially slightly softer (−13 HV) TM steel performs best, which could be explained with the beneficial influence of the tempering process.

The test conditions (load, indenter cone angle, indenter tip radius, material) influenced the transition between the different micro-mechanisms. In order to investigate the dependence of scratch characteristics on the material properties and test system characteristics, multiple linear regression models were constructed. In these models, the independent variables coming from test systems were the cone angle “γ”, the load “F_N_”, and the tip radius “*r*”, further independent variables were the material properties and the dimensionless indicators formed from them (that is in equation (2) the dependent variable Y is scratch width, and the independent variables $X_{1},X_{2},\ldots$ are the previously mentioned ones). According to this, the best fitting model of the possible ones (using the method of least squares) for the scratch width was

(3) $$2d=a_{0}+a_{1}F_{N}+a_{2}\frac{W\cdot \sigma_{y}}{E},$$

the model turned out to be statistically relevant, that is, it significantly differs from a constant function, since the F-value was 377 and *p* < 0.001. The obtained coefficients $a_{0}$, $a_{1}$ and $a_{2}$ of the respective independent variables are presented in Table 4 (see the second column).

The model with the specific coefficients takes the form

(4) $$2d=11.019+3.898F_{N}+383\cdot \frac{W\cdot \sigma_{y}}{E}.$$

For this model, the goodness-of-fit is R^2^ = 0.89. The load has the highest effect on the scratch width, while among the material parameters e.g., $\frac{W\cdot \sigma_{y}}{E}$ has some effect, this can be seen from the extent and the ranking of the absolute value of the Beta-coefficients.

The best fitting model of the possible ones for the scratch depth was

(5) $$h=a_{0}+a_{1}F_{N}+a_{2}r+a_{3}\sigma_{c},$$

the model is relevant (F = 43 and *p* < 0.001). Table 5 summarizes the coefficients of the model.

For this model, the goodness-of-fit is R^2^ = 0.572. The load has the highest effect on the scratch depth, followed by the tip radius, while among the material parameters e.g., $\sigma_{c}$ has some effect.

The best fitting model of the possible ones for the Dp was

(6) $$\mathrm{Dp}=a_{0}+a_{1}F_{N}+a_{2}r+a_{3}\gamma ,$$

which turned out to be relevant (F = 35 and *p* < 0.001). The coefficients of the model are summarized in Table 6.

For this model, the goodness-of-fit is R^2^ = 0.533. The load has the highest effect on the Dp, followed by the tip radius and the cone angle. This verified the results from Figure 5.

The best fitting model of the possible ones for the groove area (*A_g_*) was

(7) $$A_{g}=a_{0}+a_{1}F_{N}+a_{2}r+a_{3}E_{c},$$

the model is relevant since F = 51 and *p* < 0.001. The coefficients of model (6) are presented in Table 7.

For this model, the goodness-of-fit is R^2^ = 0.622. The normal load has the highest effect on the groove area, followed by the tip radius. Among the material parameters $E_{c}$ has some effect.

For the resulting wear, multiple linear regression models were constructed to see the sensitivity of material properties and test system characteristics on it. In the test systems, the sliding distance “*s*”, the load “F_N_”, the tip radius “*r*”, and the cone angle “γ” were considered as independent variables, as well as the material properties and the indicators formed from them. According to this, the best fitting model of the possible ones for the wear volume was

(8) $$V_{g}-V_{s}=a_{0}+a_{1}F_{N}+a_{2}s+a_{3}\frac{WEH}{\sigma_{M}\sigma_{Y}}+a_{4}r,$$

the model is relevant (the F-value was 57 and *p* < 0.001). The coefficients of the model are presented in Table 8.

For this model, the goodness-of-fit is R^2^ = 0.374. The load has the highest effect on the wear volume, followed by the sliding distance. In this case, the dominant influence of the load on the material loss confirms previous literature findings [20]. Among the material parameters the $\frac{WEH}{\sigma_{M}\sigma_{Y}}$ has some effect.

Based on the above discussed linear models, Table 9 summarizes the abrasive sensitivity for the resulted scratch characteristics (width, depth, degree of penetration, groove area, and volume). The factors are in increasing order of effect. The abrasive sensitivity is the extent how the independent variables (test variables and material properties and indicators formed from them) affect a dependent variable (e.g., wear) which is related to the standardized regression coefficients. Therefore, the higher the absolute value of the corresponding standardized regression coefficient is, the higher the abrasive sensitivity of the dependent variable is (wear, groove characteristics) with respect to the independent variable.

### 3.2. Pin-on-Disc Test

**Figure 7:**
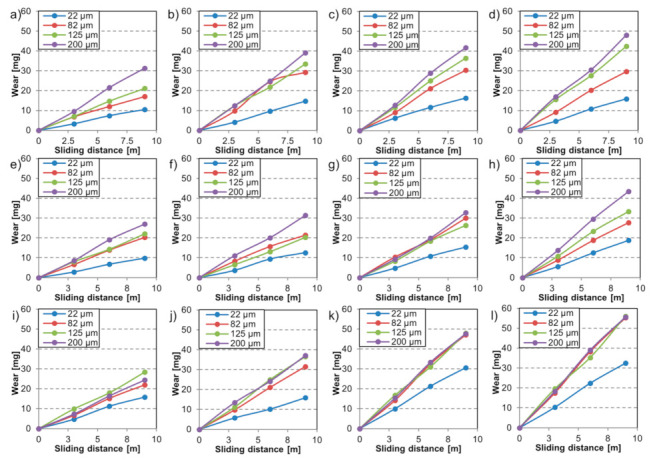
Wear (mg) in function of sliding distance for different abrasive particle sizes (22 µm, 82 µm, 125 µm, 200 µm) and increasing load (4.5 N, 11.2 N, 17.4 N, 24.2 N) for material FM (**a**–**d**), material TM (**e**–**h**), and MP steel (**i**–**l**).

The mass loss [mg] as a function of the sliding distance for the tested materials during the multi-asperity tests is shown in Figure 7. Increasing the load and the abrading particle size resulted in more severe wear.

As reported in the literature [52,53], the slope of the wear curves increases proportionally with the abrasive size up to the critical particle size (CPS). Upon entering this critical size range, the wear behavior may change [24]. This was confirmed in the tests with MP material, where the adjustment in wear rate was evident above 82 µm abrasive particle size (Figure 7i–l). The wear rate stabilized for the MP material in case of these conditions. The effect of the load was found to be more straightforward. Testing with higher load was always followed by more severe material loss.

The role of particle size and attack angle was already reported [54]. “Spike value” was introduced as a quantitative feature that takes the sharpness and size of the particle into consideration [55]. The smaller average particle size of the P800 paper would anticipate inferior material removal than on a P80 paper. However, the higher attack angle of the embedded P800 particles due to their smaller tip radius mitigates this effect. Generally, higher attack angles enhance the micro-cutting process.

**Figure 8:**
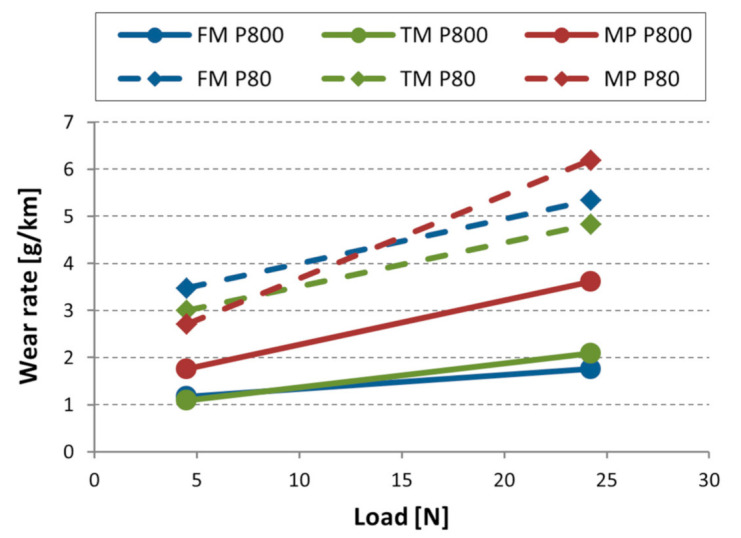
Wear rate (g/km) of tested materials with different abrasive particles (P80, P800) in the function of normal load (N).

Figure 8 shows the wear rate (g/km) in function of the normal load for the tested materials. Comparing the materials regarding wear resistance, in general, TM material performed best in front of the FM material. MP steel experienced more severe material removal. Overall, the material ranking is in line with the wear trends observed in case of the single-asperity scratch tests.

The results are influenced by the material properties originating from its microstructure and the hardness differences [56]. During the multi-asperity testing, the contact surface of the steel samples interacts with new abrasive particles when running on a spiral path inducing work hardening. For this reason, the hardness change was also investigated. In the most severe wear condition (P80 200 µm abrasive with 24.2 N load) the initial values of 478 HV for FM, 465 HV for TM, and 367 HV of MP changed to 504 HV, 488 HV, and 376 HV, respectively.

**Figure 9:**
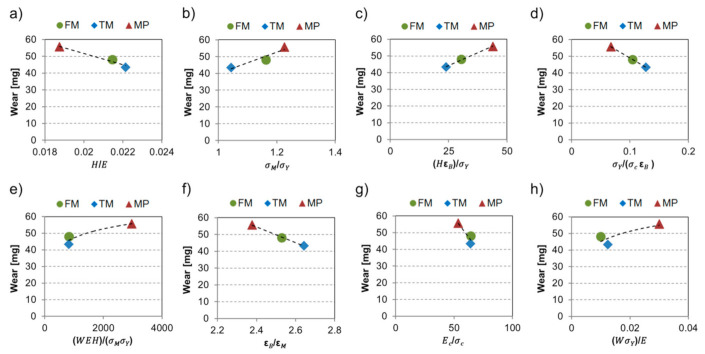
Wear of the most severe test condition (P80, 24.2 N) in the function of dimensionless features: (**a**) $\frac{H}{E}$, (**b**) $\;\frac{\sigma_{M}}{\sigma_{Y}}$, (**c**) $\frac{H\varepsilon_{B}}{\sigma_{Y}}$, (**d**) $\frac{\sigma_{Y}}{\sigma_{c}\varepsilon_{B}}$, (**e**) $\frac{WEH}{\sigma_{M}\sigma_{Y}}$, (**f**) $\frac{\varepsilon_{B}}{\varepsilon_{M}}$, (**g**) $\frac{E_{c}}{\sigma_{c}}$, (**h**) $\frac{W\sigma_{Y}}{E}$ derived from the mechanical properties.

Figure 9 shows the most severe wear condition values in function of the formed dimensionless numbers originated from the material properties. This offers an indication of the effect of derived factors on the wear trend for each material. Proportional relations were found between the material loss and $\frac{\sigma_{M}}{\sigma_{Y}}$, as well as $\frac{H\varepsilon_{B}}{\sigma_{Y}}$. Increased wear was observed by rising these dimensionless number values. In the case of $\frac{WEH}{\sigma_{M}\sigma_{Y}}$ and $\frac{W\sigma_{Y}}{E}$, a similar trend was found. Inverse relations were established between values $\frac{H}{E}$, $\frac{\sigma_{Y}}{\sigma_{c}\varepsilon_{B}}$, $\frac{\varepsilon_{B}}{\varepsilon_{M}}$, $\frac{E_{c}}{\sigma_{c}}$ and the wear of the tested steels.

Similar to the previous discussions, again multiple linear regression models are investigated to see how the resulted wear depends on the material properties as well as the testing variables. The independent variables were the parameters sliding distance “s”, load “F_N_”, and abrasive particle size “d”, together with the material properties and the dimensionless parameters derived from them. According to this, the best fitting model of the possible ones was

(9) $$\Delta m=a_{0}+a_{1}s+a_{2}d+a_{3}F_{N}\cdot +a_{4}\frac{\sigma_{y}}{\sigma_{c}\varepsilon_{B}},$$

the F-value of the model was 261 and *p* < 0.001, which again means that the model is relevant. Table 10 summarizes the coefficients of the model.

For this model, the goodness-of-fit is R^2^ = 0.848. On the wear of the test sample the time of the experiment (sliding distance) has the highest effect, furthermore, the material related parameter $\frac{\sigma_{Y}}{\sigma_{c}\varepsilon_{B}}$ has some effect.

The best fitting model of the possible ones for the change of the contact surface hardness was

(10) $$\Delta H=a_{0}+a_{1}\frac{W\sigma_{Y}}{E}+a_{2}s,$$

the model is relevant (F = 699 and *p* < 0.001). In Table 11, the coefficients of the model are presented.

For this model, the goodness-of-fit is R^2^ = 0.994. The material parameter $\frac{W\sigma_{Y}}{E}$ has a dominant effect on the hardness change, while the sliding distance also plays a role.

**Figure 10:**
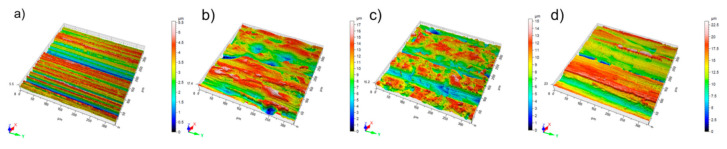
3D surface roughness of the tested materials with P80 abrasives and 24.2 N load (**a**) unworn, (**b**) FM, (**c**) TM, (**d**) MP.

The effect of wear tests resulting in the most severe wear (P80 tests with 24.2 N) was studied on the change in surface roughness parameters. To investigate the change of the surface topography, white light optical microscopy was used. Figure 10 shows the original 3D surface characteristics and their worn condition, and Table 12 summarizes the roughness values.

All the materials suffered deformation, with material deposition to form new hills and valleys. The clearest grooves were identified on the MP material indicating a dominant micro-cutting effect with continuous chip generation and material removal. The groove depth values from Figure 10 are in line with the measured wear from Figure 7.

For the surface roughness parameters as dependent variables, multiple linear regression models were built, where the independent variables were sliding distance “s”, load “F_N_”, and abrasive particle size “d”, besides the material properties and the indicators formed from them. According to this, the best fitting model for the change in Ra among the possible ones was

(11) $$\Delta Ra=a_{0}+a_{1}d+a_{2}s,$$

where the F-value of the model was 43 and *p* < 0.001, which means the model is relevant. The coefficients of the model are summarized in Table 13.

For this model, the goodness-of-fit is R^2^ = 0.493. The size of the abrasive in the experiment has the highest effect on the change in Ra of the worn surface, followed by the sliding distance. The effect of material parameters is negligible. Change in the Rz, Rt, Rp, Rv Rc, and Rq parameters follow the same trend, where the size of the abrasive particles has the highest effect on the change of the roughness parameter, followed by the sliding distance. However, the following surface roughness parameters showed different dominant dependence.

The best fitting model of the possible ones for the change in Rsm was

(12) $$\Delta \mathrm{Rsm}=a_{0}+a_{1}d+a_{2}\frac{\sigma_{y}E}{\sigma_{M}H}+a_{3}s,$$

the model is relevant (F = 30 and *p* < 0.001). The coefficients of the model are presented in detail in Table 14.

For this model, the goodness-of-fit is R^2^ = 0.431. The size of the abrasive in the experiment has the highest effect on the change in Rsm of the worn surface, while the only material parameter with some effect is $\frac{\sigma_{y}E}{\sigma_{M}H}$.

The best fitting model of the possible ones for the change in Rsk was

(13) $$\Delta \mathrm{Rsk}=a_{0}+a_{1}s+a_{2}W_{\left(20\right)}+a_{3}d,$$

the model is relevant since the F-value was 53 and *p* < 0.001. In Table 15, the coefficients of the model are presented.

For this model, the goodness-of-fit is R^2^ = 0.645. In this case, the sliding distance in the experiment has the highest effect on the change of Rsk, among the material parameters $W_{\left(20\right)}$ has some effect. The size of the abrasive particle resulted in a minor effect.

The best fitting model among the possible ones for the change in Rku was

(14) $$\Delta Rku=a_{0}+a_{1}s+a_{2}\frac{\sigma_{y}E}{\sigma_{M}H},$$

which is relevant (F = 86 and *p* < 0.001). Table 16 summarizes the coefficients of the model.

For this model, the goodness-of-fit is R^2^ = 0.658. The sliding distance in the experiment has the highest effect on the Rku of the worn surface and $\frac{\sigma_{y}E}{\sigma_{M}H}$ is the only material parameter with a significant, but mediocre effect.

According to the above-presented results it can be seen that besides the initial hardness and the Charpy impact strength the elasticity modulus and the tensile parameters play role in the change of surface parameters. These results are in line with the influence of applied loads on the micro-geometry. The micro-geometry of the moving steel surfaces under normal load suffers shear, bending, and compressive effects mainly, resulting in the appearance of plastic deformation, wedge formation, and micro-cutting [57,58].

Table 17 summarizes the findings on the discussed linear models with respect to the abrasive sensitivity for wear and the resulted surface roughness parameters. The factors that play role are presented in increasing order of effect.

### 3.3. Slurry Pot Test System

**Figure 11:**
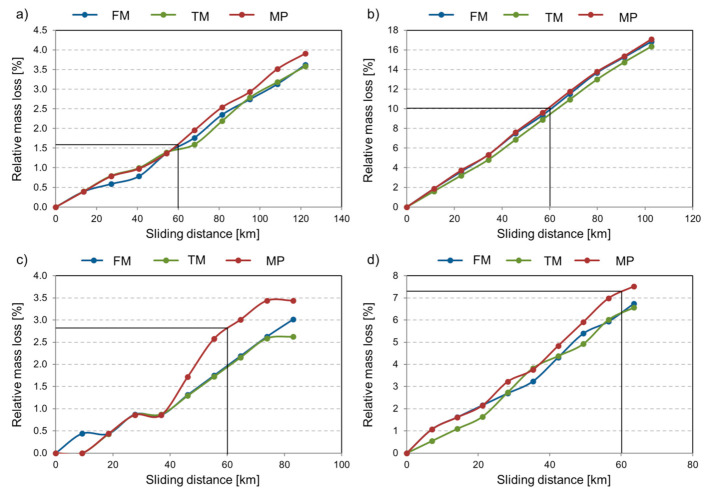
The relative mass loss (%) of the tested materials (FM, TM, MP) in the function of sliding distance in different positions; (**a**) radius 125 mm, (**b**) radius 105 mm, (**c**) radius 85 mm, (**d**) radius 65 mm.

In the slurry-pot system, the material samples move in a slurry medium at four circumferential speeds and two angles of impact and suffer abrasive erosion on the surface. The different specimen positions modified the contact areas with the slurry due to the centrifugal action of the slurry flow. For adequate comparison of wear for different radii, the contact area (specimen area exposed to wear) has been taken into account, and the values were normalized accordingly. Figure 11 shows the relative mass loss (%) as a function of sliding distance, where the initial mass was normalized with the specimen area exposed to wear. For a better comparison of the relative mass loss values, an extra vertical line is drawn at 60 km sliding distance for all radii. The standard deviation in the mass loss (%), including the repetitions, was below 0.1%. All materials showed a similar trend with a linear increase in wear. The relative wear of the specimen placed on radius 105 mm is an order of magnitude higher than the rest of the samples. Similarly, specimen mounted on radius 65 mm suffered severe wear, hence the effect of specimen orientation (angle of attack) had a more significant role on the wear severity than the difference in the radius (higher testing speed). This phenomenon could be explained by the effect of the centrifugal force on the slurry. The centrifugal force pushes the abrasive particles in a radial direction to the 45° oriented specimen surfaces, resulting in more severe material removal.

Despite the higher circumferential velocity of the slurry abrasive particles on radius 125 mm, less wear was recognized on these samples than on samples mounted on radius 85 mm (for the same orientation). This result can be attributed to the observed wear mechanisms. On specimen mounted on radius 125 mm significant pitting was also observed, except on the multiphase steel. Due to the custom design configuration of the slurry pot tester, both abrasion and erosion were co-existing. The material properties, the specimen radius, and orientation affected the wear mechanisms and the severity of the wear. In erosion literature, materials are classified as ductile or brittle based on the dependence of their erosion rate on the angle of impingement. Ductile materials have a maximum erosion rate at low angles (~15–30°), while brittle materials experience a peak erosion rate close to 90° [59]. The tested materials are considered more brittle than ductile, except the retained austenitic multiphase steel due to its microstructure and lower hardness [60]. The tests confirmed that the MP steel performed better against surface erosion, however suffered severe abrasion. As Figure 11 shows, the mass loss curves of the MP steel are clearly over the mass loss of the martensitic materials in most cases. TM was the best performing in most of the cases, closely followed by FM material.

**Figure 12:**
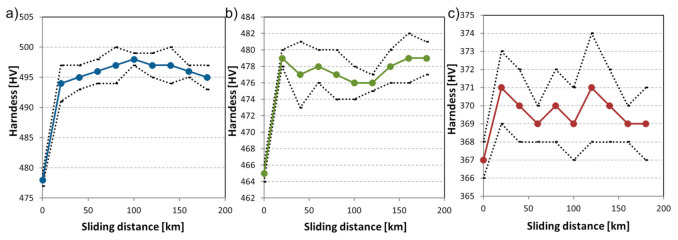
The hardness of tested slurry pot specimen in the function of wear testing time (sliding distance) (**a**) material FM, (**b**) material TM, (**c**) material MP.

Overall, TM resulted in an average mass loss of 0.0753 (%/100 km), while FM experienced more severe wear (0.0815%/100 km). The least wear-resistant multiphase steel averaged 0.0881 (%/100 km) mass loss during the slurry pot tests. The hardness of the worn zone of the specimens was monitored during the slurry pot tests. Figure 12 shows the hardness change of all tested materials in the function of the operating time.

The hardness gain was already present after the first 20 h of testing. The two best-performing martensitic materials experienced an average +15 HV hardness gain. The MP steel experienced only a minor hardening and a more significant material removal through abrasion mechanism.

Finally, similar analysis based on multiple linear regression models were carried out to study the sensitivity of material properties and test system parameters on the wear. The independent variables were the sliding distance “s”, the sliding velocity “v”, and the impact angle “α”; furthermore, the material properties and the indicators based on them. The mass loss is normalized ($\Delta m^{*}$) with the contact area to exclude the different areas of contact originating from the different radii. According to this, the best fitting model for the wear of the possible ones was

(15) $$\Delta m^{*}=a_{0}+a_{1}s+a_{2}\alpha +a_{3}v,$$

since F = 65 and *p* < 0.001, it means the model is relevant. The coefficients of it are presented in Table 18.

For this model, the goodness-of-fit is R^2^ = 0.626. The sliding distance has the highest influence on the wear of the test sample, followed by the impact angle and the velocity. The effect of material parameters is negligible. The model confirmed the less dominant role of the impact velocity compared to the impact angle (as concluded from Figure 11) in this test system.

The best fitting model among the possible ones for the hardness gain was

(16) $$\Delta H=a_{0}+a_{1}\frac{W\sigma_{Y}}{E}+a_{2}s+a_{3}\frac{E_{c}}{\sigma_{c}},$$

the model is relevant (F-value was 8959 and *p* < 0.001). The coefficients of the model are presented in detail in Table 19.

For this model, the goodness-of-fit is R^2^ = 0.996. From the material parameters $\frac{W\sigma_{Y}}{E}$ had the highest effect on the hardness change. The compression strength and modulus ($\frac{E_{c}}{\sigma_{c}}$) also played a role as well as the time of the experiment (sliding distance).

**Figure 13:**
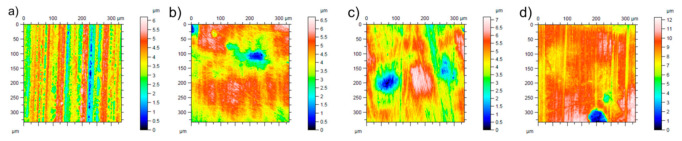
2D surface roughness of the tested materials from radius 105 mm, 45° after 11.4 km run (**a**) unworn, (**b**) FM, (**c**) TM, (**d**) MP.

The change in the surface topography of the tested specimens was also investigated with an optical profilometer (Figure 13). Before testing, the surface roughness parameters of all specimens were similar due to the same manufacturing process. After testing, surface roughness values, which are shown in Table 20, of the specimen resulted in the most severe wear condition. The surface of the contact area roughened and also experienced minor pitting due to the abrasive erosion. TM material surface roughened most indicating a less effective material removal through abrasive polishing, which was confirmed by the mass loss plots (Figure 11).

For all cases, the results are polished surfaces in different degrees with micro-cut grooves indicating abrasion, as well as co-existing micro-pits in different severity due to the abrasive erosion.

Similarly to the previous discussions, multiple linear regression models were evaluated to see the dependence of the surface roughness parameters on the duration of the test (sliding distance), the velocity of the test specimen, the contact angle, and the material properties. Since the roughness parameters of the examined materials have a high deviation, therefore for most of the models the goodness-of-fit varied in a large extent (between 0.27 and 0.72) and mostly, only one explanatory variable had a significant effect. Change in the surface roughness parameters of Ra, Rt, Rz, Rp, Rv Rc, Rq, and Rsm followed the same trend, where the sliding distance in the experiment has the highest effect on the change of the roughness parameter, e.g., the best fitting model among the possible ones for Rt was

(17) $$\Delta \mathrm{Rt}=a_{0}+a_{1}s,$$

the model is relevant (F = 83 and *p* < 0.001). Table 21 summarizes the coefficients of the model.

For this model, the goodness-of-fit is R^2^ = 0.782. The sliding velocity in the experiment has the highest effect on the Rt. The effect of other parameters was negligible. However, the Rsk surface roughness parameter showed different results. The best fitting model among the possible ones for Rsk was

(18) $$\Delta \mathrm{Rsk}=a_{0}+a_{1}v+a_{2}s,$$

the model is relevant since F = 7 and *p* < 0.001. The coefficients of the model are presented in Table 22.

For this model, the goodness-of-fit is R^2^ = 0.401. The sliding velocity in the experiment has the highest effect on the Rsk of the worn surface followed by the sliding distance.

Table 23 summarizes the abrasive sensitivity for wear, hardness change, and the resulted surface roughness parameters based on the results of the above discussed linear models. The factors are presented in increasing order of effect.

## 4. Conclusions

This study investigated the abrasive wear behavior of newly developed martensitic and multiphase steels and enabled the ranking of them in different abrasion conditions. Three different test systems represented a broad range of the abrasion wear mechanism, simulating different material responses to the complex appearance of abrasion in the targeted wear parts. Comparing the test systems, the tested materials ranked similarly based on their wear performance, however in each configuration, the dominant variable of the wear mechanism differed.

The significance and contributions of test parameters, extended material properties, and the dimensionless features formed from them were investigated on the wear behavior and the surface deformation. Correlation between parameters was obtained by multiple linear regression models.

In terms of wear resistance, the tempered martensitic steel performed best across all the investigated test systems, closely followed by the fresh martensitic structured material. The multiphase steel resulted in the most severe material loss.

- The normal load was the driving factor in the material removal in the abrasive scratch tests, followed by the sliding distance. The dimensionless number $\frac{WEH}{\sigma_{M}\sigma_{Y}}$ had a mediocre effect, while the indenter tip radius had a slight influence on the wear mechanism.
- The dominant variable affecting the surface topography of the materials in the scratch tests was found to be the normal load, followed by the tip radius. However, sensitivity to the material’s compression properties $E_{c}$, $\sigma_{c}$ was found in case of groove depth and groove area. The width of the scratch was influenced by the parameter $\frac{W\sigma_{y}}{E}$.
- In the abrasive pin-on-disc test system, the sliding distance and the abrasive particle size were found to be dominant on the wear severity, followed by the normal load. From the material parameters $\frac{\sigma_{y}}{\sigma_{c}\varepsilon_{B}}$ had a slight effect.
- Considering the worn surface topography, the particle size was found to be the dominant shaping factor followed by the sliding distance. The effect of $\frac{\sigma_{y}E}{\sigma_{M}H}$ on the microgeometry was clear in the case of the parameters Rsm and Rsk.
- Considering the hardness change, the parameter $\frac{W\sigma_{Y}}{E}$ was found to have a significant effect, the model describing the hardness change resulted an R^2^ value of 0.994.
- Proportional relations between the wear values of the materials and the dimensionless numbers of $\frac{\sigma_{M}}{\sigma_{Y}}$, as well as $\frac{H\varepsilon_{B}}{\sigma_{Y}}$ were established in case of the most severe wear testing conditions (P80, 24.2 N).
- There are reciprocal relations between values $\frac{H}{E}$, $\frac{\sigma_{Y}}{\sigma_{c}\varepsilon_{B}}$, $\frac{\varepsilon_{B}}{\varepsilon_{M}}$, $\frac{E_{c}}{\sigma_{c}}$ and the wear of the materials. Increasing these dimensionless number values resulted in lower wear.
- In the slurry pot test system, the sliding speed and the impact angle was found to have a significant effect on the wear, followed by the sliding distance. The effect of material factors was negligible in this case.
- Considering the surface deformation, only the sliding distance appeared to have a significant effect on the worn surface topography.
- Specimen experienced hardening of the abraded contact surface, which remained approximately constant afterward. Sensitivity analysis proved that for the hardness gain, the variable $\frac{W\sigma_{Y}}{E}$ had a significant influence. The material’s compression properties ($E_{c}$, $\sigma_{c}$) were also found to have an effect.

Mathematical models were developed based on experimental results in different complexity test systems to determine the relationship between the wear, change of surface parameters and the test variables, material properties, and dimensionless features formed from them. This investigation aids the design of an optimized tribo-system by enabling the prediction of the wear process in complex abrasive environment for the investigated steels.

## Figures and Tables

**Table 1:** Chemical composition of the tested materials [wt%].

Material	%C	%Mn	%Si	%P	%S	%Ti	%Cr	%Ni	%B	%Mo
	(Max)	(Max)	(Max)	(Max)	(Max)	(Max)	(Max)	(Max)	(Max)	(Max)
FM	0.2	1.9	0.5	0.02	0.005	0.05	0.5	-	0.004	-
TM	0.2	1.9	0.5	0.02	0.005	0.05	0.5	-	0.004	-
MP	0.2	1.6	-	0.018	0.005	-	1.9	~0.20	-	0.4

**Table 2:** Properties of the tested steels.

Properties	Notation	Unit	FM	TM	MP
Microstructure	-	-	Fresh martensite	Tempered martensite	Multiphase steel with retained austenite
Hardness	H	HV	478	465	367
Ultimate tensile strength	σ_M_	MPa	1528	1474	1264
Yield strength	σ_Y_	MPa	1314	1413	1031
Young modulus	E	GPa	217	206	192
Uniform elongation	ε_M_	%	3.4	2.8	5.3
Fracture elongation	ε_B_	%	8.6	7.4	12.6
Charpy ISO 148-1 (20 °C/−40 °C)	W	J	28/17	38/21	179/32
Compressive strength	σ_c_	MPa	1460	1501	1213
Compressive modulus	E_c_	GPa	94	96	65

**Table 3:** Slurry pot test parameters.

Radius	(mm)	125	105	85	65
Surface velocity	(m/s)	1.885	1.583	1.282	0.98
Angle	(°)	135	45	135	45
Wear area	(%)	55	75	25	20
Sliding distance	(m/20h)	13,572	11,400	9229	7057

**Table 4:** Coefficients of the regression model for scratch width in single-asperity test.

Model	Coefficient	Standardized Regression Coefficient, Beta	t	*p*
Constant	11.019	-	7.594	<0.001
F_N_	3.898	0.922	26.829	<0.001
$\frac{W\cdot \sigma_{y}}{E}$	383	0.202	5.871	<0.001

**Table 5:** Coefficients of the regression model for scratch depth in single-asperity test.

Model	Coefficient	Standardized Regression Coefficient, Beta	t	*p*
Constant	5.951		2.932	<0.001
F_N_	0.448	0.662	9.867	<0.001
r	−0.014	−0.350	−5.215	<0.001
$\sigma_{c}$	−0.003	−0.157	−2.344	<0.001

**Table 6:** Coefficients of the regression model for Dp in single-asperity scratch test.

Model	Coefficient	Standardized Regression Coefficient, Beta	t	*p*
Constant	0.213	-	4.384	<0.001
F_N_	0.013	0.545	7.647	<0.001
r	−0.001	−0.448	−6.289	<0.001
γ	−0.001	−0.188	−2.633	<0.001

**Table 7:** Coefficients of the regression model for groove area for single-asperity scratch test.

Model	Coefficient	Standardized Regression Coefficient, Beta	t	*p*
Constant	185.217	-	3.961	<0.001
F_N_	19.837	0.693	10.813	<0.001
r	−0.513	−0.300	−4.675	<0.001
$E_{c}$	−0.002	−0.229	−3.575	<0.001

**Table 8:** Coefficients of the regression model for groove volume in single-asperity scratch test.

Model	Coefficient	Standardized Regression Coefficient, Beta	t	*p*
Constant	−90,251.994	-	–5.305	<0.001
F_N_	16,474.646	0.459	11.292	<0.001
s	38.693	0.300	7.389	<0.001
$\frac{WEH}{\sigma_{M}\sigma_{Y}}$	30.409	0.213	5.248	<0.001
r	−361.825	−0.168	–4.142	<0.001

**Table 9:** Abrasive sensitivity ranking to single-asperity scratch system variables.

Effect of influencing factors	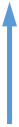	F_N_	-	-	-	F_N_
			F_N_	F_N_	F_N_	s
			r	r	r	$\frac{WEH}{\sigma_{M}\sigma_{Y}}$
		$\frac{W\sigma_{y}}{E}$	$\sigma_{c}$	γ	$E_{c}$	r
Independent variable		*2d*	*h*	*Dp*	*A_g_*	*V_g_*

**Table 10:** Coefficients of the regression model for multi-asperity test.

Model	Coefficient	Standardized Regression Coefficient, Beta	t	*p*
Constant	−0.002	-	−1.209	<0.001
s	0.003	0.827	29.031	<0.001
d	0.0001	0.268	9.404	<0.001
F_N_	0.0005	0.241	8.450	<0.001
$\frac{\sigma_{y}}{\sigma_{c}\varepsilon_{B}}$	−0.101	−0.184	−6.468	<0.001

**Table 11:** Coefficients of the regression model for multi-asperity test.

Model	Coefficient	Standardized Regression Coefficient, Beta	t	*p*
Constant	539.836	-	156.937	<0.001
$\frac{W\sigma_{Y}}{E}$	−5904.081	−0.986	−37.018	<0.001
s	1.722	0.145	5.436	0.001

**Table 12:** Surface characteristics after the wear tests (P80 with 24.2 N) and the change in %.

Parameter	Name	Unit	FM	%	TM	%	MP	%
Ra	Arithmetical mean height	(µm)	2.650	+111	2.446	+94	2.757	+104
Rz	Max. height	(µm)	14.257	+66	14.721	+80	16.075	+88
Rp	Highest peak	(µm)	6.517	+43	6.319	+45	7.511	+78
Rv	Lowest valley	(µm)	7.740	+91	8.402	+120	8.565	+98
Rt	Total height	(µm)	19.578	+63	20.675	+91	22.415	+91
Rc	Average height of profile element	(µm)	9.608	+79	9.514	+91	10.748	+109
Rq	Root mean square height	(µm)	3.261	+98	3.096	+91	3.487	+103
Rsm	Mean width of profile elements	(µm)	201.838	+16	215.626	+42	245.867	+68
Rsk	Skewness	-	−0.216	−173	−0.348	−155	−0.277	+190
Rku	Kurtosis	-	2.740	−70	3.405	0	3.300	−1

**Table 13:** Coefficients of the regression model for multi-asperity test.

Model	Coefficient	Standardized Regression Coefficient, Beta	t	*p*
Constant	0.715	-	6.642	<0.001
d	0.005	0.527	6.981	<0.001
s	0.067	0.453	6.000	<0.001

**Table 14:** Coefficients of the regression model for multi-asperity test.

Model	Coefficient	Standardized Regression Coefficient, Beta	t	*p*
Constant	373.585	-	8.320	<0.001
d	0.152	0.450	5.982	<0.001
$\frac{\sigma_{y}E}{\sigma_{M}H}$	−5.497	−0.391	−5.202	<0.001
s	1.816	0.370	4.922	<0.001

**Table 15:** Coefficients of the regression model for multi-asperity test.

Model	Coefficient	Standardized Regression Coefficient, Beta	t	*p*
Constant	0.334	-	10.381	<0.001
s	−0.029	−0.640	−10.0682	<0.001
$W_{\left(20\right)}$	−0.001	−0.423	−6.659	<0.001
d	−0.001	−0.247	−3.891	<0.001

**Table 16:** Coefficients of the regression model for multi-asperity test.

Model	Coefficient	Standardized Regression Coefficient, Beta	t	*p*
Constant	7.164	-	11.926	<0.001
s	−0.057	−0.715	−11.527	<0.001
$\frac{\sigma_{y}E}{\sigma_{M}H}$	−0.085	−0.373	−6.022	<0.001

**Table 17:** Abrasive sensitivity ranking to pin-on-disc test system features.

Effect of influencing factors	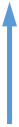	s	$\frac{W\sigma_{Y}}{E}$	-	-	-	-
		d			d	s	s
		F_N_	s	d	$\frac{\sigma_{y}E}{\sigma_{M}H}$	*W_(20)_*	-
		$\frac{\sigma_{y}}{\sigma_{c}\varepsilon_{B}}$	*-*	s	s	d	$\frac{\sigma_{y}E}{\sigma_{M}H}$
Independent variable		Δm	ΔH	Ra, Rz, Rp, Rv, Rt, Rc, Rq	Rsm	Rsk	Rku

**Table 18:** Coefficients of the regression model for slurry pot test.

Model	Coefficient	Standardized Regression Coefficient, Beta	t	*p*
Constant	−0.854	-	−1.157	<0.001
s	0.00001	0.476	7.891	<0.001
α	−0.045	−0.680	−10.709	<0.001
v	3.336	0.378	5.663	<0.001

**Table 19:** Coefficients of the regression model for slurry pot test.

Model	Coefficient	Standardized Regression Coefficient, Beta	t	*p*
Constant	7541.733	-	12.438	<0.001
$\frac{W\sigma_{Y}}{E}$	−77,274.537	−1.275	−16.060	<0.001
s	0.001	0.030	4.965	<0.001
$\frac{E_{c}}{\sigma_{c}}$	−30.240	−0.279	−3.510	<0.001

**Table 20:** Surface roughness parameters of the tested materials after 180 h testing on radius 105 mm with 45° impact angle and the change in %.

Parameter	Name	Unit	FM	%	TM	%	MP	%
Ra	Arithmetical mean height	(µm)	0.382	+5	0.4185	+24	0.410	+21
Rz	Max. height	(µm)	1.744	+4	1.929	+27	1.903	+23
Rp	Highest peak	(µm)	0.889	+8	0.9835	+29	0.987	+27
Rv	Lowest valley	(µm)	0.855	+1	0.946	+24	0.916	+19
Rt	Total height	(µm)	6.928	+184	12.0945	+483	10.763	+398
Rc	Average height of profile element	(µm)	1.215	−3	1.533	+30	1.42	+17
Rq	Root mean square height	(µm)	0.476	+6	0.5165	+27	0.506	+23
Rsm	Mean width of profile elements	(µm)	347.327	+3	354.292	+1	345.168	−2
Rsk	Skewness	-	0.108	−418	0.1055	+254	0.133	+359
Rku	Kurtosis	-	2.489	+1	2.5205	+5	2.508	+2

**Table 21:** Coefficients of the regression model for slurry pot test.

Model	Coefficient	Standardized Regression Coefficient, Beta	t	*p*
Constant	2.504	-	3.592	<0.001
s	0.001	0.890	9.132	<0.001

**Table 22:** Coefficients of the regression model for slurry pot test.

Model	Coefficient	Standardized Regression Coefficient, Beta	t	*p*
Constant	0.233	-	3.371	<0.001
v	−0.155	−0.555	−3.203	<0.001
s	0.001	0.452	2.609	0.001

**Table 23:** Abrasive sensitivity ranking to slurry pot system variables.

Effect of influencing factors	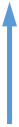	-	$\frac{W\sigma_{Y}}{E}$	s	-
		v		-	v
		α	s	-	s
		s	$\frac{E_{c}}{\sigma_{c}}$	-	-
Independent variable		Δm *	ΔH	Ra, Rz, Rp, Rv, Rt, Rc, Rq, Rsm, Rku	Rsk

## Data Availability

The data presented in this study are available on request from the corresponding author. The data are not publicly available due to the present work was carried out within the framework of a project that is bound by Intellectual Property constraints.

## Nomenclature

**Notation**	**Name**	**Unit**
Al_2_O_3_	Aluminium oxide	
Analysis of variance	ANOVA	
ASTM	American Society for Testing and Materials	
CPS	Critical particle size	
FM	Fresh martensitic	
ISO	International Organization for Standardization	
MLP	Multilayer perceptrons	
MoB	Molybdenum boride	
MP	Multiphase	
RBF	Radial basis networks	
SiC	Silicon carbide	
TM	Tempered martensitic	
$\Delta m^{*}$	Normalized mass loss with contact area	%
2d	Scratch width	µm
A_g_	Groove area	µm^2^
A_s_	Shoulder/ridge area	µm^2^
d	Abrasive size	µm
Dp	Degree of penetration	-
E	Young modulus	GPa
E_c_	Compressive modulus	GPa
F_N_	Normal load	N
h	Scratch depth	µm
H	Hardness	HV
k	Specific wear rate	mm^3^/Nm
r	Tip radius	µm
Ra	Arithmetical mean height	µm
Rc	Profile element average height	µm
Rku	Kurtosis	-
Rp	Highest peak	µm
Rq	Root mean square height	µm
Rsk	Skewness	-
Rsm	Mean width of the profile elements	µm
Rt	Total height of the profile	µm
Rv	Lowest valley	µm
Rz	Max. height	µm
s	Sliding distance	m
v	Velocity	m/s
V_g_	Groove volume	mm^3^
V_s_	Shoulder/ridge volume	mm^3^
W	Charpy ISO 148-1 (20 °C/−40 °C)	J
α	Attack angle/impact angle	°
β	Degree of wear	-
ε_B_	Fracture elongation	%
ε_M_	Uniform elongation	%
σ_c_	Compressive strength	MPa
σ_M_	Ultimate tensile strength	MPa
σ_Y_	Yield strength	MPa
γ	Indenter cone angle	°

## References

[1] Kalácska Á., De Baets P., Fauconnier D., Schramm F., Frerichs L., Sukumaran J. (2020). Abrasive wear behaviour of 27MnB5 steel used in agricultural tines. Wear.

[2] Rendón J., Olsson M. (2009). Abrasive wear resistance of some commercial abrasion resistant steels evaluated by laboratory test methods. Wear.

[3] Xie Y., Jiang J., Tufa K.Y., Yick S. (2015). Wear resistance of materials used for slurry transport. Wear.

[4] National Research Council Canada, Associate Committee on Tribology (1986). A Strategy for Tribology in Canada: Enhancing Reliability and Efficiency Through the Reduction of Wear and Friction.

[5] Ahmed S., Thakare O.P., Shrivastava R., Sharma S., Sapate S.G. (2018). A Review on Slurry Abrasion of Hard Faced Steels. Mater. Today Proc..

[6] Sapate S.G., Chopde A.D., Nimbalkar P.M., Chandrakar D.K. (2008). Effect of microstructure on slurry abrasion response of En-31 steel. Mater. Des..

[7] Bootle M. Wear in rotodynamic (centrifugal) slurry pumps. Proceedings of the Calgary Pump Symposium.

[8] Javaheri V., Porter D., Kuokkala V.-T. (2018). Slurry erosion of steel—Review of tests, mechanisms and materials. Wear.

[9] Camacho J., Lewis R., Dwyer-Joyce R.S. (2009). Solid particle erosion caused by rice grains. Wear.

[10] Muhandes H., Kalácska Á., Székely L., Keresztes R., Kalácska G. (2020). Abrasive Sensitivity of Engineering Polymers and a Bio-Composite under Different Abrasive Conditions. Materials.

[11] Májlinger K., Kalácska G., Orbulov I., Zsidai L., Bozóki B., Keresztes R. (2016). Global Approach of Tribomechanical Development of Hybrid Aluminium Matrix Syntactic Foams. Tribol. Lett..

[12] Sidorov S.A., Khoroshenkov V.K., Lobachevskii Y.P., Akhmedova T.S. (2017). Improving Wear Resistance of Agricultural Machine Components by Applying Hard-Alloy Thick-Layer Coatings Using Plasma Surfacing. Metallurgist.

[13] Bayer R.G. (2004). Mechanical Wear Fundamentals and Testing.

[14] Blau P.J. (1992). ASM Handbook, Volume 18—Friction, Lubrication, and Wear Technology.

[15] Xu X., Ederveen F.H., van der Zwaag S., Xu W. (2016). Correlating the abrasion resistance of low alloy steels to the standard mechanical properties: A statistical analysis over a larger data set. Wear.

[16] Khruschov M.M. (1974). Principles of abrasive wear. Wear.

[17] Zum Gahr K.-H. (1987). Microstructure and Wear of Materials. https://www.elsevier.com/books/microstructure-and-wear-of-materials/zum-gahr/978-0-444-42754-0.

[18] Nathan G.K., Jones W.J.D. (1966). The empirical relationship between abrasive wear and the applied conditions. Wear.

[19] Goddard J., Wilman H. (1962). A theory of friction and wear during the abrasion of metals. Wear.

[20] Zambrano O.A., Aguilar Y., Valdés J., Rodríguez S.A., Coronado J.J. (2016). Effect of normal load on abrasive wear resistance and wear micromechanisms in FeMnAlC alloy and other austenitic steels. Wear.

[21] Ma X., Liu R., Li D.Y. (2000). Abrasive wear behavior of D2 tool steel with respect to load and sliding speed under dry sand/rubber wheel abrasion condition. Wear.

[22] Mulhearn T.O., Samuels L.E. (1962). The abrasion of metals: A model of the process. Wear.

[23] Larsen-Badse J. (1968). Influence of grit size on the groove formation during sliding abrasion. Wear.

[24] Gåhlin R., Jacobson S. (1999). The particle size effect in abrasion studied by controlled abrasive surfaces. Wear.

[25] Sin H., Saka N., Suh N.P. (1979). Abrasive wear mechanisms and the grit size effect. Wear.

[26] Gahr K.H.Z. (1988). Modelling of two-body abrasive wear. Wear.

[27] Mutton P.J., Watson J.D. (1978). Some effects of microstructure on the abrasion resistance of metals. Wear.

[28] Sundström A., Rendón J., Olsson M. (2001). Wear behaviour of some low alloyed steels under combined impact/abrasion contact conditions. Wear.

[29] Deng X., Wang Z., Tian Y., Fu T., Wang G. (2013). An investigation of mechanical property and three-body impact abrasive wear behavior of a 0.27% C dual phase steel. Mater. Des..

[30] Modi O.P., Mondal D.P., Prasad B.K., Singh M., Khaira H.K. (2003). Abrasive wear behaviour of a high carbon steel: Effects of microstructure and experimental parameters and correlation with mechanical properties. Mater. Sci. Eng. A.

[31] Aksoy M., Karamiş M.B., Evin E. (1996). An evaluation of the wear behaviour of a dual-phase low-carbon steel. Wear.

[32] Jha A.K., Prasad B.K., Modi O.P., Das S., Yegneswaran A.H. (2003). Correlating microstructural features and mechanical properties with abrasion resistance of a high strength low alloy steel. Wear.

[33] Xu X., van der Zwaag S., Xu W. (2016). The effect of martensite volume fraction on the scratch and abrasion resistance of a ferrite–martensite dual phase steel. Wear.

[34] Chen W., Biswas S., Roberts A., O’Shea J., Williams K. (2017). Abrasion wear resistance of wall lining materials in bins and chutes during iron ore mining. Int. J. Miner. Process..

[35] Aydin F. (2021). The investigation of the effect of particle size on wear performance of AA7075/Al2O3 composites using statistical analysis and different machine learning methods. Adv. Powder Technol..

[36] Tsai W., Humphrey J.A.C., Cornet I., Levy A.V. (1981). Experimental measurement of accelerated erosion in a slurry pot tester. Wear.

[37] Rahiman A.H.S., Smart D.S.R., Wilson B., Ebrahim I., Eldhose B., Mathew B., Murickan R.T. (2020). Dry sliding wear analysis OF Al5083/CNT/Ni/MoB hybrid composite using DOE Taguchi method. Wear.

[38] Bustillo A., Yu Pimenov D., Matuszewski M., Mikolajczyk T. (2018). Using artificial intelligence models for the prediction of surface wear based on surface isotropy levels. Robot. Comput. Integr. Manuf..

[39] Matuszewski M., Słomion M., Mazurkiewicz A., Yu D. Pimenov: Mathematical models of changes in the surface layer of frictional pairs as a tool to optimize the wear process. Proceedings of the MATEC Web of Conferences.

[40] ASTM G171-03(2017) (2009). Standard Test Method for Scratch Hardness of Materials Using a Diamond Stylus.

[41] Lindroos M., Valtonen K., Kemppainen A., Laukkanen A., Holmberg K., Kuokkala V.-T. (2015). Wear behavior and work hardening of high strength steels in high stress abrasion. Wear.

[42] Kayaba T., Hokkirigawa K., Kato K. (1986). Analysis of the abrasive wear mechanism by successive observations of wear processes in a scanning electron microscope. Wear.

[43] Hokkirigawa K., Kato K. (1988). An experimental and theoretical investigation of ploughing, cutting and wedge formation during abrasive wear. Tribol. Int..

[44] Hokkirigawa K., Kato K., Li Z.Z. (1988). The effect of hardness on the transition of the abrasive wear mechanism of steels. Wear.

[45] Woldman M., Van Der Heide E., Tinga T., Masen M.A. (2013). The influence of abrasive body dimensions on single asperity wear. Wear.

[46] ASTM G132-96(2013) (2013). Standard Test Method for Pin Abrasion Testing.

[47] Matuszewski M., Mikolajczyk T., Pimenov D.Y., Styp-Rekowski M. (2017). Influence of structure isotropy of machined surface on the wear process. Int. J. Adv. Manuf. Technol..

[48] Silicon Carbide Paper, Grit 120 32 mm (1¼") dia. 100 pcs. (40400129). https://e-shop.struers.com/CA/EN/products/Miscellaneous/Non-destructive_Testing/Silicon_Carbide_Paper_Grit_120_32_mm_.

[49] Macchini R., Bradley M.S.A., Deng T. (2013). Influence of particle size, density, particle concentration on bend erosive wear in pneumatic conveyors. Wear.

[50] Szabadi L. (2011). Abrasive Wear of Multilayer Hot-Dip Galvanized Coatings. Ph.D. Thesis.

[51] Mezlini S., Zidi M., Arfa H., Ben Tkaya M., Kapsa P. (2005). Experimental, numerical and analytical studies of abrasive wear: Correlation between wear mechanisms and friction coefficient. Comptes Rendus Mécanique.

[52] Coronado J.J., Sinatora A. (2011). Effect of abrasive size on wear of metallic materials and its relationship with microchips morphology and wear micromechanisms: Part 1. Wear.

[53] Adamiak M. (2012). Abrasion Resistance of Materials.

[54] Huffington J.D. (1978). Abrasion groove sizes and shapes in relation to the mechanism of abrasion. Wear.

[55] Hamblin M.G., Stachowiak G.W. (1995). A multi-scale measure of particle abrasivity, and its relation to two-body abrasive wear. Wear.

[56] Owsiak Z. (1997). Wear of symmetrical wedge-shaped tillage tools. Soil Tillage Res..

[57] Bhushan B. (2000). Modern Tribology Handbook.

[58] Stachowiak G.W., Batchelor A.W. (2014). Engineering Tribology.

[59] Kosel T.H. (1962). Solid particle erosion. Brit. J. Appl. Phys..

[60] Song C., Wang H., Sun Z., Wei Z., Yu H., Chen H., Wang Y., Lu J. (2020). Effect of multiphase microstructure on fatigue crack propagation behavior in TRIP-assisted steels. Int. J. Fatigue.

